# Musculoskeletal Pain and Brain Morphology: Oxytocin’s Potential as a Treatment for Chronic Pain in Aging

**DOI:** 10.3389/fnagi.2019.00338

**Published:** 2019-12-13

**Authors:** Désirée Lussier, Yenisel Cruz-Almeida, Natalie C. Ebner

**Affiliations:** ^1^Department of Psychology, University of Florida, Gainesville, FL, United States; ^2^Pain Research and Intervention Center of Excellence, University of Florida, Gainesville, FL, United States; ^3^Claude D. Pepper Older American Independence Center, Institute on Aging, University of Florida, Gainesville, FL, United States; ^4^Department of Clinical and Health Psychology, Center for Cognitive Aging and Memory, University of Florida, Gainesville, FL, United States; ^5^Department of Community Dentistry & Behavioral Science, College of Dentistry, University of Florida, Gainesville, FL, United States; ^6^Departments of Aging & Geriatric Research, Epidemiology and Neuroscience, College of Medicine, University of Florida, Gainesville, FL, United States

**Keywords:** oxytocin, aging, chronic pain, brain morphology, treatment, older adults

## Abstract

Chronic pain disproportionately affects older adults, severely impacting quality of life and independent living, with musculoskeletal pain most prevalent. Chronic musculoskeletal pain is associated with specific structural alterations in the brain and interindividual variability in brain structure is likely an important contributor to susceptibility for the development of chronic pain. However, understanding of age-related structural changes in the brain and their associations with chronic musculoskeletal pain is currently limited. Oxytocin (OT), a neuropeptide present in the periphery and central nervous system, has been implicated in pain attenuation. Variation of the endogenous OT system (e.g., OT receptor genotype, blood, saliva, and cerebrospinal fluid OT levels) is associated with morphology in brain regions involved in pain processing and modulation. Intranasal OT administration has been shown to attenuate pain. Yet, studies investigating the efficacy of OT for management of chronic musculoskeletal pain are lacking, including among older individuals who are particularly susceptible to the development of chronic musculoskeletal pain. The goal of this focused narrative review was to synthesize previously parallel lines of work on the relationships between chronic pain, brain morphology, and OT in the context of aging. Based on the existing evidence, we propose that research on the use of intranasal OT administration as an intervention for chronic pain in older adults is needed and constitutes a promising future direction for this field. The paper concludes with suggestions for future research in the emerging field, guided by our proposed *Model of Oxytocin’s Anagelsic and Brain Structural Effects in Aging.*

## Introduction

Pain constitutes a multidimensional experience that includes cognitive, affective, and sensory characteristics in response to noxious stimuli. Multiple systems for classifying pain exist. These include multidimensional classification systems, such as the International Association for the Study of Pain (IASP) Classification of Chronic Pain, and systems based on a single dimension of the pain experience (Merskey and Bogduk, 1994). Of the latter systems, those based on underlying pathophysiology and pain duration (i.e., acute vs. chronic pain) are most commonly used and form the basis of this review. A more recent comprehensive definition further supports the distinction between acute and chronic pain while accounting for the significant emotional distress or functional disability resulting from pain (e.g., interference with activities of daily life and participation in social roles). *For an in-depth review relating to pain definitions and etiologies*, see Treede et al. (2015).

Acute pain is generally defined in terms of its duration and is usually accompanied by relatively high levels of pathology that resolves with healing of the underlying injury. Acute pain, often accompanied by protective reflexes (e.g., withdrawal of damaged limb, muscle spasm), serves an important biological function by warning of the potential for or extent of injury. Thus, acute pain serves a necessary protective function to ensure survival. In contrast, chronic pain is recognized as pain that extends beyond the period of healing, with low levels of identified pathology that are often insufficient to explain the presence and/or the extent of the clinical pain presentation (Treede et al., 2015).

Chronic pain is persistent pain that “*disrupts sleep and normal living, ceases to serve a protective function, and instead degrades health and functional capability*” (Chapman and Stillman, 1996). Unlike acute pain, our current understanding suggests that chronic pain serves no adaptive purpose. While there are multiple subtypes of chronic pain (e.g., headache, fibromyalgia, and pelvic pain), chronic musculoskeletal pain is the most prevalent with back pain afflicting the highest proportion of patients (Traeger et al., 2016; Murphy et al., 2017). In addition to this, the various pain subtypes are characterized by somewhat varying morphological brain signatures, although with some overlap (e.g., reduced gray matter density in the insula) (Baliki et al., 2011). This paper will, therefore, primarily focus on chronic musculoskeletal pain, when possible.

Chronic pain is a costly problem plaguing global public health (Goldberg and McGee, 2011). In the U.S. alone, an estimated 126.1 million adults reported experiencing pain within a 3-month time frame, with 25.3 million reporting their pain as chronic (i.e., having daily pain 3 months or longer) (Nahin, 2015). The financial cost of pain to the U.S. economy is estimated to be as high as $635 billion in medical care and productivity loss annually (Gereau et al., 2014), with the risk of chronic pain conditions, particularly related to chronic musculoskeletal pain, increasing with age (Murphy et al., 2017). In a national survey, 34% of older adults reported being troubled by pain, while 22% reported their pain as significantly impacting daily activities (Shi et al., 2010). Over half of respondents in another survey of U.S. adults over the age of 65 reported being bothered by pain within the last month (Patel et al., 2013). This is particularly concerning given that older adults comprise the fastest growing population segment in the United States (US Census Bureau, 2017).

The current opioid crisis highlights the need for alternative treatments for chronic pain that are both safe and effective (Volkow and Collins, 2017). Oxytocin (OT), a neuropeptide that naturally occurs in the body and the brain, may constitute a promising candidate. There is growing evidence of safe and effective administration of the neuropeptide intranasally (Born et al., 2002; MacDonald et al., 2011). OT is involved in pain modulation and single-dose OT administration studies support its role in pain attenuation (Boll et al., 2017). Paloyelis et al. (2016a) make the argument that there is evidence intranasal OT may be modulating pain via its effects on neuronal activity and cerebral blood flow alterations (Paloyelis et al., 2016b) in regions implicated in pain processing (Treede et al., 1999; Peyron et al., 2002). Additionally, a separate line of research suggests that interindividual variation in the endogenous OT system (e.g., OT receptor (*OXTR*) genotype, OT blood, saliva, and cerebrospinal fluid (CSF) levels) is associated with morphological variation in the same brain regions that are involved in the pain experience. However, the specific relationships between chronic pain, brain morphology, and OT in the context of aging are currently not understood.

**FIGURE 1:**
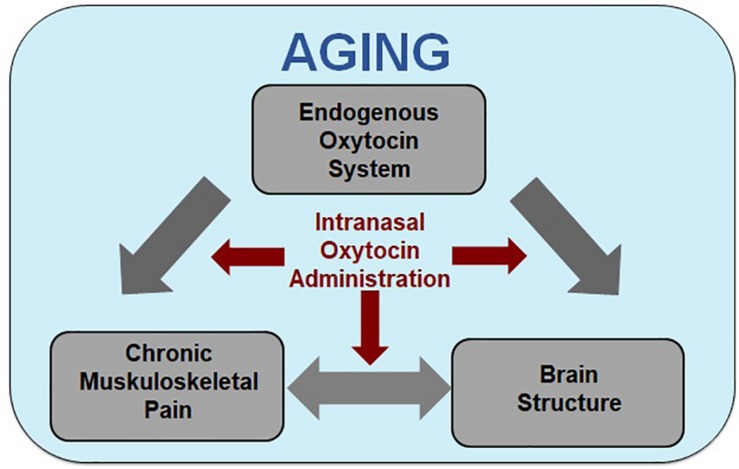
Model of oxytocin’s analgesic and brain structural effects in aging. Increased age is associated with increased incidence of susceptibility for the development of chronic pain, alterations in brain morphology in regions associated with pain processing, and potential changes to the endogenous oxytocin (OT) system. Although the direction and mechanisms of the effects are largely unknown, there is evidence of two-way interactions between chronic muskuloskeletal pain and brain structure, with the endogenous OT system potentially affecting both. Our model integrates the associations between chronic muskuloskeletal pain, brain structure (e.g., gray matter morphology and white matter integrity), and the endogenous OT system [e.g., circulatory levels, OT receptor (OXTR) genotype and expression, OT binding site locations] within the context of aging and proposes that the three may interact to increase age-related chronic pain susceptibility. Based on these interactions, we propose intranasally administered OT as a treatment for chronic pain in older individuals constitutes a promising research direction.

This focused narrative review addresses an existing gap in the literature by synthesizing studies that support associations of chronic musculoskeletal pain, given its high prevalence, with brain morphology in regions that also constitute critical targets of intranasal OT administration in aging. The literature basis on chronic pain, brain morphology, and OT is very limited, with no study specifically addressing the use of OT for pain treatment in older adults. Given the lack of a specific literature on the effects of OT on the pain experience and pain processing in aging, a systematic review or a metanalysis was not possible at this time. Instead, we developed our proposal of the beneficial effects of intranasal OT administration for pain alleviation in aging based on an integrative review of currently parallel lines of research. In particular, we summarized the existing evidence related to: (i) associations between chronic musculoskeletal pain and brain morphology, (ii) age-related changes in brain morphology in pain processing regions, (iii) associations between the endogenous OT system and brain structure, (iv) associations between the endogenous OT system and pain, (v) effects of OT administration on pain in humans, (vi) OT mechanisms underlying pain attenuation and efficacy in animals, and (vii) effects of OT in aging. We integrated these parallel literatures to derive our newly proposed *Model of Oxytocin’s Analgesic and Brain Structural Effects in Aging* (Figure 1). We discuss the strengths and weaknesses of the existing research and identify current knowledge gaps, and, guided by our novel framework, we conclude with suggestions for future directions and clinical applications of this emerging research field.

**FIGURE 2:**
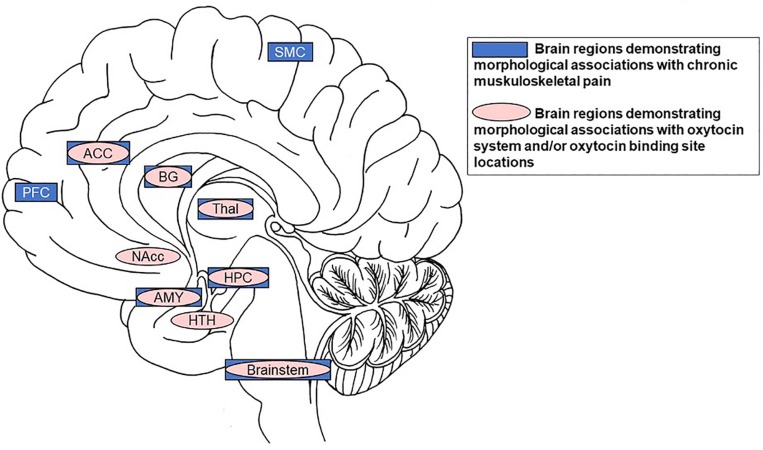
Summary of significant overlap between brain regions demonstrating morphological associations with chronic muskuloskeletal pain (dark boxes) and those demonstrating morphological associations with the OT system and/or OT binding site locations (lighter ovals). Specifically, the anterior cingulate cortex, basal ganglia, thalamus, hippocampus, amygdala, and brainstem are volumetrically associated with chronic musculoskeletal pain and contain receptors/binding-sites with high affinity for OT. Notes: SMC = motor/somatosensory cortex; ACC = anterior cingulate cortex; BG = basal ganglia; PFC = prefrontal cortex; Thal = thalamus; Nacc = nucleus accumbens; HPC = hippocampus; AMY = amygdala; HTH = hypothalamus.

The specific focus of this paper is on older individuals, given their susceptibility to chronic pain (Murphy et al., 2017). The high prevalence of chronic pain in older adults combined with census bureau projections that adults over 65 years are the fastest growing population segment in industrialized nations render the development of effective treatment solutions particularly relevant among this age group. Current research on the efficacy of intranasal OT administration in older adults is still scarce (but see Campbell et al., 2014; Ebner et al., 2016, 2015; Grainger et al., 2018a; Horta et al., 2018) and has not yet been applied to chronic pain. In light of the opioid crisis (Volkow and Collins, 2017), we propose that intranasal OT as a treatment for chronic pain in older individuals constitutes a promising research direction. This proposal is based on evidence of a significant overlap between brain regions relevant in pain processing with brain regions identified as crucial targets of the OT system (Figure 2).

## Methods

To determine the article basis for this focused narrative review, we conducted a search to extract peer-reviewed articles published in 2018 or earlier. The initial literature review was conducted between January 2018 and August 2018 by the first author, with updates to the literature as we became aware of new articles until time of submission. Databases included PubMed and Google Scholar. We began the search by using combined search terms (“aging” or “older adults” and “oxytocin” and “chronic pain” and/or “musculoskeletal pain”). This search, however, yielded no studies that contained all search criteria terms. We therefore extended the search by leveraging parallel lines of research using the above search criteria in different combinations of pairs or individually. To capture the literature on brain morphology and its interactions with pain and OT, we included the search terms “brain morphology” and/or “brain structure” and/or “brain volumes.”

Articles were identified based on the relevance of the title, followed by a review of the full abstract. A full-text review of those articles that passed the first two criteria followed. We also searched the reference section of relevant articles to identify additional articles for inclusion. In particular, articles were added to the final selection if they met at least one of the following criteria: (1) focused on or included aging or older adults and one of the other original search criteria, (2) focused on one or more of the original search criteria plus one of the search criteria related to brain morphology, and included human subjects. Animal literature and literature involving acute experimental pain were included in instances where human or chronic pain literature was lacking. Articles were excluded if: (1) they were not specifically related to chronic pain, OT, or aging, or if (2) they were focused on chronic pain and brain morphology but not musculoskeletal pain specifically.

## Literature Review

No previous article directly addressed the effects of OT on pain in older individuals in relation to brain morphology. However, 135 articles addressed the following four topics, which structured our literature review: (1) “Chronic Musculoskeletal Pain and Structural Brain Alterations”, (2) “Oxytocin’s Role in Pain Modulation”, (3) “Endogenous Oxytocin and Brain Structure”, (4) “Oxytocin and the Aging Brain”.

### Chronic Musculoskeletal Pain and Structural Brain Alterations

Chronic musculoskeletal pain is the most prevalent chronic pain subtype in older individuals (Patel et al., 2013; Murphy et al., 2017). Additionally, morphological brain signatures vary by chronic pain subtype (Baliki et al., 2011). This section, therefore, is focused on studies of brain morphology and chronic musculoskeletal pain.

Alterations in brain structure associated with chronic musculoskeletal pain are widespread. For example, cortical gray matter volume was lower in 26 chronic back pain patients, over half of which had a musculoskeletal diagnosis accounting for the pain, with overall gray matter decreases of 1.3 cm^3^ each year of reported chronic pain compared to age and gender matched controls (Apkarian et al., 2004). Ung et al. (2014), using structural MRI scans of 47 patients, with ages ranging from 19 to 60 years old, and 47 gender and age matched controls successfully classified 76% of chronic back pain patients based on gray matter density of the prefrontal, somatosensory, motor, and visual cortices, temporal lobe, amygdala, medial orbital gyrus, and cerebellum using multivariate support vector analysis. Furthermore, chronic musculoskeletal pain patients exhibited volumetric reductions, compared to pain-free age-matched controls, in the dorsolateral prefrontal, motor (Bishop et al., 2018), somatosensory (Schmidt-Wilcke et al., 2006; Bishop et al., 2018), and cingulate cortices, with gray matter density reductions in the dorsolateral prefrontal and the middle cingulate cortices (Ivo et al., 2013).

Regarding subcortical structures, the basal ganglia (Schmidt-Wilcke et al., 2006), particularly the caudate (Bishop et al., 2018), showed higher volumes related to chronic musculoskeletal pain. The thalamus showed larger gray matter volume (Schmidt-Wilcke et al., 2006), with lower bilateral gray matter density (Ivo et al., 2013), in chronic pain compared to controls. Brainstem volumetric reductions were associated with increased self-reported pain intensity and unpleasantness levels (Schmidt-Wilcke et al., 2006). Results regarding the hippocampus, however, were mixed. While one study found lower hippocampal volume, mediated by cortisol levels, in chronic back pain patients than controls (Vachon-Presseau et al., 2013), another study found higher amygdala and hippocampus gray matter volumes associated with chronic musculoskeletal pain (Bishop et al., 2018).

Increases in subcortical volumes along with decreases in frontal volumes may be reflective of the shift in pain processing from nociceptive to emotional regions and circuits in the transition from acute to chronic pain (Hashmi et al., 2013). However, not everyone who suffers an acute injury develops chronic pain. Lower back pain became chronic for approximately 19–30% of patients with acute lower back injury (Traeger et al., 2016). Additionally, individuals who habituated to experimental application of repeated noxious heat stimulus over 11 days did not show gray matter density changes compared to baseline and those who sensitized (Stankewitz et al., 2013).

There is also evidence of altered gray and white matter microstructure (i.e., fractional anisotropy) of the medial prefrontal cortex, the lateral prefrontal cortex, and the nucleus accumbens, along with connecting fibers (e.g., superior longitudinal fasciculus), as predictors of the transition to chronic pain for patients with a single back pain episode (Mansour et al., 2013). Chronic musculoskeletal pain patients also demonstrated altered white matter densities and connectivity within the dorsal attention network (Bishop et al., 2018). Such structural alterations may underlie the functional changes that predicted low back pain chronification in a prospective study (Baliki et al., 2012). Of note, most of the current work on brain morphology and musculoskeletal pain is in young to middle-aged individuals or comprises mixed age populations with a relatively small number of older adults. Only very few studies to date have focused on older adults, as summarized next.

#### Associations Between Susceptibility to Chronic Pain and Age-Related Structural Brain Changes

Age-related structural brain changes may underlie the increased prevalence of chronic pain in older adults. Aging is associated with volumetric decrease in regions involved in pain processing and modulation including the prefrontal cortex (Farrell, 2012), hippocampus (Raz et al., 2005; Farrell, 2012), caudate (Raz et al., 2005), and to a lesser extent the brainstem (Farrell, 2012). Older adults with chronic low back pain showed reduced white matter cingulate and left posterior parietal gray matter volumes (Buckalew et al., 2008). Further, middle-aged and older adults with unresolved chronic pain showed gray matter reductions in the cingulate, prefrontal, motor, and premotor cortices (Ruscheweyh et al., 2011) compared to age-matched controls.

Age-related changes in white matter integrity may also play a role in chronic pain susceptibility with advanced age. In particular, multiple white matter fiber tracts showed reduced integrity, reflected in lower fractional anisotropy, and higher mean diffusivity, with increasing age (Westlye et al., 2010; Sala et al., 2012). Community-dwelling older adults with chronic pain had lower whole-brain fractional anisotropy (Cruz-Almeida et al., 2017) and higher white matter hyperintensity burden compared to age-matched controls (Buckalew et al., 2008; Cruz-Almeida et al., 2017). Recent evidence also suggested that chronic pain was associated with added “age-like” brain atrophy in relatively healthy older individuals using whole-brain measures of gray and white matter volume (Cruz-Almeida et al., 2019), further supporting associations between pain and brain morphology in aging.

#### Normalization of Brain Structure After Chronic Pain Treatment

The literature debates whether brain alterations observed among various chronic pain conditions are subsequent to or precede the pain, with evidence for both. For example, chronic osteoarthritis pain patients, who had volumetric reductions in the dorsolateral prefrontal and anterior cingulate cortices, insula, operculum, amygdala, and brainstem, showed an increase in these regions after successful pain alleviation by hip replacement surgery (Rodriguez-Raecke et al., 2009). Patients who received surgery that successfully alleviated their chronic back pain showed a normalization of dorsolateral prefrontal, primary motor, and insula cortical thickness that was associated with pain decrease (Seminowicz et al., 2011). Additionally, experimentally induced volumetric alterations in the cingulate and somatosensory cortices via eight consecutive days of thermal pain application in healthy young adults was reversible with pain cessation (Teutsch et al., 2008). In contrast, Mansour et al. (2013) reported that underlying structural differences predisposed patients to pain chronification after a low back injury. While the mechanisms underlying normalization of brain changes in chronic pain are likely complex and multifactorial, it is plausible that there are predisposing and/or protective factors (i.e., age, genetics, environment, and life experience), which interact with pain-induced specific brain alterations.

Regardless of the source or pathophysiology of the pain, patients often gain only partial relief. Current common treatment options for chronic pain include systemic analgesics (e.g., non-steroidal anti-inflammatories (NSAIDs), antidepressants, opiates, anticonvulsants, alpha-adrenergic receptor antagonists), topical analgesics, and surgery. However, most of these regimens are limited by tolerability issues and potential complications. NSAID use is associated with increased cardiovascular and gastrointestinal risks (Schmidt et al., 2016). Opiate pain-relievers carry a high risk for addiction, are known to depress CNS function, and they are currently recommended as a last resort for pain relief (Schneiderhan et al., 2017). Surgery is also not a viable option for everyone (Brummett et al., 2015). Furthermore, older adults are more susceptible to side effects of these treatments due to age-related changes in physiology, increased comorbidities, and enhanced risks for medication interactions (Wallace and Paauw, 2015). This prompts the need for novel, low-side effect treatments for chronic pain management in older adults. In consideration of literature supporting OT’s analgesic effects, as reviewed next, we propose that intranasal OT constitutes a promising candidate for the management of chronic pain, via mechanisms that impact brain morphology in regions associated with chronic pain.

### Oxytocin’s Role in Pain Modulation

Oxytocin is a regulatory neuropeptide that is synthesized in the magnocellular cells of the paraventricular (PVN) and supraoptic nuclei (SON) of the hypothalamus and is released diffusely in the CNS by magnocellular dendrites (Gimpl and Fahrenholz, 2001; Ludwig and Leng, 2006; Lee et al., 2009; Insel, 2010; Bethlehem et al., 2013) and neuronal projections to regions of the basal ganglia and the limbic system (Landgraf and Neumann, 2004; Knobloch et al., 2012; Grinevich et al., 2016). OT has widespread, context-dependent biological and behavioral functions (Carter, 2014). Pain processing regions that show altered morphology in chronic pain overlap with brain regions that constitute crucial targets of the OT system (Figure 2). Evidence suggests that OT is involved in modulation of the pain experience (Rash et al., 2013; Tracy et al., 2015; Boll et al., 2017). This section will discuss (1) “Endogenous Oxytocin and Chronic Pain” (2) “Exogenous Oxytocin Administration and Pain” and (3) Potential Mechanisms for Oxytocin’s Role in Pain Attenuation.

#### Endogenous Oxytocin and Chronic Pain

Although care must be taken to not infer direct causation from this particular line of research, some studies have shown a correlation between endogenous OT levels and chronic pain. For example, a study of 40 children suffering from recurrent abdominal pain showed lower (24 pmol/l) endogenous blood plasma OT levels than 34 age and sex matched controls (63 pmol/l) (Alfven et al., 1994). Furthermore, 32 individuals with migraine showed differential unpleasantness responses to capsaicin-induced pain, while elevated OT plasma levels during pain experience correlated with lower pain-related distress (You et al., 2018).

Determination of endogenous OT levels to date, however, faces challenges given the highly invasive nature of determining CSF OT concentrations via spinal tap and the controversial literature regarding the correlation between CSF and saliva/blood plasma OT levels. While some work suggests that blood plasma OT levels may be indicative of CSF levels in humans (Carson et al., 2015), typically peripheral samples (blood or saliva) are obtained as proxy for central levels. There is also some work that suggests that saliva OT levels may constitute a more reliable marker of central concentrations than blood plasma levels (Martin et al., 2018), acknowledging, however, that if attempting to measure OT levels after exogenous OT administration measures based on saliva samples may be biased due to nasal drip.

#### Exogenous Oxytocin Administration and Pain

Evidence on effects of intranasal OT on pain management is limited and mixed, with close to no research on the long-term use for chronic pain management specifically. Therefore, we supplemented our review on the efficacy of OT for chronic pain management with evidence of effects of intranasal OT administration on acute experimentally induced pain specifically in humans. While this section attempted to be comprehensive with regard to human studies investigating OT administration on chronic pain, report of studies investigating the effects of OT administration on experimentally induced pain was limited to those studies focused on intranasal OT administration. Additional discussion of research across the translational continuum or spectrum (e.g., animal or *in vitro* studies), furthermore, provides a brief overview of the mechanistic evidence for OT’s analgesic effects.

##### Chronic pain

The majority of studies examining the effect of OT on chronic pain used single-dose administration to determine efficacy for temporary pain relief. For example, in a single case-study a terminally ill cancer patient suffering from intractable thoracic pain experienced 88% pain reduction that lasted approximately 77 min after intraventricular single-dose OT administration (Madrazo et al., 1987). Furthermore, patients with low back pain experienced temporary analgesia after single-dose (0.172 μg/kg for chronic and 0.121 μg/kg in acute) intrathecal OT administration in humans (Yang, 1994). Intravenous OT, compared to placebo, also increased visceral perception pressure thresholds in patients with painful irritable bowel syndrome at continuous infusion rates of 20 mU/min or greater (maximum of 50 mU/min), with no effect at 10mU/min (Louvel et al., 1996). It should be noted that the intravenous route of administration may not be acting on the same mechanism as centrally administered OT. Additionally, none of these early studies evaluated the effects of intranasal or repeated OT administration.

A more recent study which administered daily intranasal OT (80 IU) over a 3-week period did not provide therapeutic results for women with fibromyalgia (Mameli et al., 2014). However, it has been speculated that concomitant use of NSAIDs may have interfered with OT’s analgesic effects in this study (Kwong and Chan, 2015), as 12 of the 14 participants were taking NSAIDs concurrently. Additionally, the mean age of the sample was 51.9 (±7.8) indicating that many of the participants were middle-aged or older. The potential for NSAIDs to interfere with the effectiveness of intranasal OT for chronic pain management in this specific study warrants further investigation. However, it is also possible that intranasal OT may not provide the same benefits for older women as for younger women and older men, as suggested by emerging evidence of sex and/or age variations in intranasal OT response (Campbell et al., 2014; Ebner et al., 2015, 2016; Grainger et al., 2018a, b; Horta et al., 2018).

##### Experimentally induced acute pain

Single-dose administration in the context of acute experimental pain induction has been primarily conducted in generally healthy young adults and supports OT’s pain-modulatory role. In an acute single-dose study with 20 young men, 32 IU intranasal OT administered 45 min prior to scanning reduced amygdala activation in response to painful electrical stimuli to the hand compared to placebo (Singer et al., 2005). In 13 healthy younger men, 40 IU intranasal OT modulated neural responses, as evoked potentials, to perception of laser-induced pain that selectively targeted the epidermis Aδ- and C-fibers when compared to placebo (Paloyelis et al., 2016a, b). 40 IU intranasal OT also increased conditioned pain modulation using a cold pressor task and reduced anxiety in 30 healthy young adults (Goodin et al., 2014). In contrast, a 32 IU single-dose intranasal OT administration study did not find an effect on heat pain ratings or neural processing in 30 healthy younger men, but did see an effect of emotional context on pain unpleasantness ratings in the OT group compared to placebo (Zunhammer et al., 2016).

Supplementing the limited evidence in humans, animal studies confirm that single-dose OT administration attenuates acute pain. For example, rats who were administered OT directly to the trigeminal ganglion showed a relief of whisker pad mechanical hypersensitivity resulting from infraorbital nerve damage (Kubo et al., 2017). In an animal model of pain associated with mild traumatic brain injury, intranasal OT alleviated reactive as well as spontaneous ongoing non-reactive pain for up to 4 h (Meidahl et al., 2017). PVN stimulation in rats further decreased withdrawal responses to both mechanical and cold stimuli to the paw, with effects that disappeared when an *OXTR* antagonist was administered (Miranda-Cardenas et al., 2006). However, OT’s efficacy for pain relief may act in a dose-dependent manner. In particular, adult male rats who were injected with a low-dose of OT intravenously showed reduced C-type nociceptor mediated action potentials, while higher doses of injected OT had a pro-nociceptive effect (Juif and Poisbeau, 2013).

#### Potential Mechanisms for Oxytocin’s Role in Pain Attenuation

Oxytocin may exert its effect on pain via multiple peripheral and central mechanisms (Grinevich et al., 2016). Most of what is known about the mechanisms by which OT exerts its pain attenuating effect comes from animal work. However, given similarities in binding site locations across species, animal work can be leveraged to inform possible mechanisms of action in humans. The following section briefly discusses select proposed mechanisms of OT’s pain attenuation. These include peripheral and direct actions on nerve fibers, effects on emotional state and reduction of pain-related anxiety, interactions with the endogenous opioid system, as well as reductions in inflammation, and neuroprotective properties. This section provides a brief overview of potential ways OT may exert its pain attenuating effects, some of which relate to or may impact brain morphology.

##### Peripheral and direct nerve effects

In the periphery, localized OT may act on Aδ- and C-fibers to inhibit or reduce pain. For example, primary afferent fiber immunofluorescence indicates the presence of *OXTR* in nociceptive skin terminals in humans (González-Hernández et al., 2017). Additionally, direct administration of OT to the trigeminal ganglion resulted in mechanical hypersensitivity relief and local increase in vasopressin receptor (*V1AR*) immunoreactive neuron innervations in the whisker pads of rats with infraorbital nerve injury (Kubo et al., 2017). In rats, localized peripheral OT administration inhibited nociceptive Aδ and C fiber activity in the spinal cord dorsal horn (González-Hernández et al., 2017; García-Boll et al., 2018), an effect that was eliminated by an *OXTR*, but not a *V1AR*, antagonist (García-Boll et al., 2018). Juif and Poisbeau (2013) observed C-type nociceptor action potential reduction with low-dose (<5 μg) intravenous OT administration but pronociceptive effects at higher doses in spinal cord neurons of anesthetized male rats. This dose-related effect was also seen in Yang (1994), who found that 0.067 μg/kg induced pain relief but that higher doses (0.067 μg/kg) induced ataxia in rats. In another study in rats, intrathecal, but not intravenous, OT administration increased pain tolerance and *OXTR* concentrations in the brain stem and glutamatergic pathways (Nessren et al., 2017). Thus, intravenous versus intrathecal (peripheral vs. central) administration may have different effects in animals. This is also consistent with human studies, wherein side-effect profiles (MacDonald et al., 2011; United States Library of Medicine, 2019) vary by administration route. However, research into potential differences in administration-route effects for pain is limited in humans, warranting a potential future direction for investigation. Additionally, effects may vary by dosage, with higher doses not further increasing efficacy for pain relief but instead reducing it. *For a comprehensive review of animal studies on OT and pain* see Rash et al. (2013).

##### Effects on pain-related emotional states

Oxytocin’s pain attenuating effects may be, to some extent, due to the neuropeptide’s effect on emotional state and the alleviation of pain-related anxiety. As discussed above, single-dose intranasal OT administration in young men reduced amygdala activation to painful stimuli delivered to the hand (Singer et al., 2005), possibly indicative of reduction in pain-related anxiety. At the same time, there was no effect on empathy-related insular activation when observing their partners receiving a painful stimulus. These results were primarily driven by individuals who scored as “selfish” (*n* = 6), versus “prosocial” (*n* = 12; with 2 unable to be categorized) in a monetary economic game. In another study, intranasal OT resulted in greater effects of emotional picture content on subjective thermal pain levels in healthy young men (Zunhammer et al., 2016). Participants in the intranasal OT condition, compared to controls, rated pain unpleasantness levels higher when viewing images of negative emotional valence and lower when image valence was positive. Intranasal OT administration in healthy young adults also reduced anxiety and negative mood associated with a pain modulation task (Goodin et al., 2014).

Furthermore, chronic pain is often comorbid with disorders of mood (McWilliams et al., 2003), such as depression and anxiety, with several mechanisms potentially underlying these associations. Chronic pain may also share common alterations in brain morphology. For example, basal ganglia volumes were correlated with aspects of major depressive disorder (Lacerda et al., 2003) as well as chronic pain. Thus the emotional valence and anxiety-reducing potential of intranasal OT could be leveraged in the context of long-term chronic pain management and is a promising direction for future investigation.

##### Interactions with the opioid system

There is evidence that OT interacts with the opioid system during pain alleviation (Yang, 1994; Zubrzycka et al., 2005; Taati and Tamaddonfard, 2017) which may, at least partially, underlie OT’s pain-alleviating function. For example, intrathecal OT increased spinal-cord levels of beta-endorphin, L-encephalin, and dynorphin A1-13 with analgesia in both human patients with acute and chronic low back pain as well as in rats (Yang, 1994). Microinjection of OT to the ventrolateral orbital cortex increased paw withdrawal thresholds to mechanical stimuli in rats (Taati and Tamaddonfard, 2017). These pain attenuating effects of OT, however, were diminished or eliminated with the application of an *OXTR* or opioid receptor antagonist (Yang, 1994; Taati and Tamaddonfard, 2017). Additionally, OT administration decreased opioid tolerance in animal models (Kovács et al., 1985; Kriván et al., 1992). However, Louvel et al. (1996) did not find a reduction in intravenous OT efficacy with administration of naloxone, an intranasally administered opioid antagonist, emphasizing effects of OT administration route. This may potentially indicate varying methods of action when delivered peripherally versus centrally, given that centrally administered OT was rendered ineffective by centrally administered opioid antagonists, but peripherally administered OT was not. Taken together, these findings suggest the potential for intranasal OT as concomitant therapy to reduce the need for opioid drugs. They also suggest that explicit care must be taken to avoid opioid-related complications that might arise from concurrent use of both OT and opioid pain-relievers (e.g., the potential for abuse and/or increased chances of accidental overdose).

##### Anti-inflammatory effects

Pain-relieving effects may also be due to OT’s anti-inflammatory and neuroprotective properties. For example, reduced neuroendocrine and cytokine activation to bacteria in 10 healthy young and middle-aged men in a placebo-controlled cross-over design in which intravenous OT (1 pmol⋅kg^–1^⋅min^–1^) or saline, with and without bacterial lipopolysaccharides, was administered for 90 min (Clodi et al., 2008). Furthermore, *OXTR* expression may be, at least partially, driven by proinflammatory cytokine presence. For example, treatment with 1 ng/ml of the cytokine protein interleukin-1β for 30 min resulted in *OXTR* mRNA up-regulation and an increase in *OXTR* expression in human amnion epithelial cells (Terzidou et al., 2011). In a study investigating wound-healing and circulatory OT, participants in the upper quartile ranges of OT concentration showed faster experimenter-induced blister healing than those in the lowest quartile OT concentration (Gouin et al., 2010). However, OT levels were not predictive of changes in cytokine presence [interleukin-6 or tumor necrosis factor-α (TNF-α)] in this study, while vasopressin was predictive of TNF-α levels. Given that neuroinflammation is associated with central sensitization and chronic pain (Ji et al., 2019), neuroinflammation likely contributes to some of the pain-related brain morphological alterations. Additionally, neuroinflammation is associated with both negative mood states and chronic opiate use in chronic pain patients, implying a very close connection between the mechanisms discussed in this section. *For a more comprehensive review on neuroinflammation, pain, affect and opioid use* see Cahill and Taylor (2017).

##### Neuroprotective properties

Animal studies have also demonstrated neuroprotective effects of OT. For example, male rats treated with intranasal OT, compared to placebo, showed a reduction in necrotic lesion in an animal model of ischemic stroke (Etehadi Moghadam et al., 2018). Similarly, there is evidence of neuroplastic effects in rats, after 2 weeks of 1 mg/kg OT administration, as seen in hippocampus cell proliferation, differentiation, and dendritic complexity increases as compared to placebo (Sánchez-Vidaña et al., 2016). *For a more comprehensive review on OT’s neuroprotective properties* see Vargas-Martínez et al. (2014).

In summary, intranasal OT appears to exert its pain-attenuating effects via multiple, though closely linked, mechanisms, some of which are also related to brain morphology in regions involved in pain processing. At this point, the direction of effects and whether effects are direct or indirect cannot be conclusively determined. Thus, future research is warranted with the goal of confirming mechanistic processes underlying OT’s effect on pain alleviation, including in aging.

### Endogenous Oxytocin and Brain Structure

We propose that the effects of OT on pain processing and modulation may be related to mechanisms affecting brain morphology in key regions of the endogenous OT system that overlap with pain processing regions (Figure 2). The following section briefly discusses what is currently known about the endogenous OT system and brain structure.

#### Associations Between Blood Plasma and Saliva Oxytocin Levels and Brain Morphology

There is evidence that plasma OT levels were negatively correlated with right amygdala and right hippocampal volumes in 30 adults (Andari et al., 2014) and 33 women, 25 of whom had experienced early life maltreatment (Mielke et al., 2018). In addition, in the same study plasma OT levels were positively correlated with nucleus accumbens volume in women who had not experienced early life maltreatment. These findings suggest that endogenous OT levels are associated with morphology in brain regions that also show alterations in chronic pain.

#### Associations Between Oxytocin Genotype and Brain Morphology

The *OXTR* genotype, found on the human chromosome 3p25 (Simmons et al., 1995; Meyer-Lindenberg et al., 2011), is associated with brain morphology in regions that demonstrate alterations in chronic pain. Two single nucleotide polymorphisms (SNP), rs53576 and rs2254298, show a relationship with brain volumes, often in association with behavioral phenotype and environmental factors. For example, the rs53576 G-allele was associated with ventral striatum gray matter volume alterations in response to early life experiences (Dannlowski et al., 2016). Volumes of the amygdala, superior parietal lobule, temporal pole, and frontal regions were furthermore correlated with insecure childhood attachment for GG homozygotes but not for A-allele carriers (Schneider-Hassloff et al., 2016). Additionally, the *OXTR* rs2254298 A-allele genotype was positively associated with amygdala volume in Japanese adults (Inoue et al., 2010). Adult GG homozygote men, compared to A-allele carriers, showed thinner cortical gray matter in the dorsal anterior cingulate (Wang et al., 2017). However, there is evidence that adolescent girls with homozygous G alleles had greater overall gray matter and dorsomedial anterior cingulate volume but smaller amygdala and brainstem volumes than A-allele carriers (Furman et al., 2011).

#### Central Nervous Oxytocin Binding Sites

Oxytocin binds to multiple g-coupled protein receptors, sharing some binding site overlap with vasopressin although often with opposing actions (Stoop, 2012). This section discusses OT binding sites, which include *V1AR* and *OXTR* sites, with *OXTR* having a much higher affinity for OT than receptors for vasopressin. Much of what is known about *OXTR* and OT binding site locations comes from post-mortem human and animal studies.

While there are some key differences, there is also overlap in *OXTR* expression concentrations in the CNS across species (Insel, 2010), including non-human primates (Freeman and Young, 2016). Rogers et al. (2018), for example, found OT immunoreactive fibers in the straight gyrus of the orbitofrontal cortex and anterior cingulate gyrus, a region involved in pain processing, in both humans and chimpanzees, but not macaques. Immunohistological investigation of the ventral hypothalamus of the macaque showed the presence of *OXTR* in the septal nucleus and preoptic area (Boccia et al., 2001). Using a combination of autoradiography and *in situ* hybridization, *OXTR* expression was observed in the Meynert nucleus basalis, pedunculopontine tegmental nucleus, superior colliculus, trapezoid body, and ventromedial hypothalamus of the rhesus macaque (Freeman et al., 2014). The common marmoset showed strong OT binding in the nucleus accumbens, thalamic nucleus, motor trigeminal nucleus, and spinal trigeminal nucleus substantia gelatinosa, with less, although still present, binding in the putamen, the diagonal band of Brocca, superior colliculus, inferior olive nucleus, and vagal dorsal motor (Schorscher-Petcu et al., 2009). Rodent CNS binding sites for OT, however, were found primarily in regions related to olfactory processing, including the medial amygdala (Ferguson et al., 2001). *For a more extensive review on OT animal models and their applicability to human physiology and behavior* see Freeman and Bales (2018).

Post-mortem studies in humans show an extensive distribution of binding sites with an affinity for OT throughout regions of the brain relevant to pain processing and modulation. Using *in vitro* light microscope autoradiography, OT binding sites were observed throughout the supper spinal cord and brainstem of 8 male and 4 female brains, with ages ranging from 40 to 81 years old (Loup et al., 1989), as well as in the nucleus basalis of Meynert, vertical limb of the diagonal band of Broca, lateral septal nucleus, hypothalamus, globus pallidus, and ventral pallidum (Loup et al., 1991). However, caution must be taken regarding interpretability of these two studies due to radioligand *V1AR* cross-reactivity. Monoclonal antibody staining has shown presence of *OXTR* in the amygdala, medial preoptic area, hypothalamus, olfactory nucleus, vertical limb of the diagonal band, ventrolateral septum, anterior cingulate, and hypoglossal and solitary nuclei in two female adult brains, ages 28 and 44 (Boccia et al., 2013). A very recent study (Quintana et al., 2019) found *OXTR* expression pathway networks in subcortical and olfactory regions, including the pallidum, caudate, putamen, and thalamus in the brains of 5 male and 1 female adults (mean age 42.5 ± 11.2 years).

Thus, combining human and animal work, there is strong evidence of associations between the endogenous OT system (*OXTR* genotype, binding-site expression, blood and CSF levels) and brain morphology in regions that are also involved in pain processing and modulation. However, the generalizability of these findings to aging is currently unknown.

### Oxytocin and the Aging Brain

Much of what is known about OT in human aging comes from post-mortem work. Only very recently have studies started to investigate the effects of intranasal OT administration in older adults (Ebner et al., 2013; Huffmeijer et al., 2013), with mixed findings. Next, the research available on age-related changes in the endogenous OT system and exogenous OT administration in older adults is summarized.

#### Age-Related Changes in the Oxytocin System

Animal models of age-related changes in the endogenous OT system have generated mixed results. For example, radioimmunoassay did not identify differences in OT plasma levels for rats ranging in age from 3 to 32 months (Fliers and Swaab, 1983). Additionally, immunocytochemically identified OT fiber tracts showed no differences between 5 and 34-month old rats in density within the locus coeruleus, solitary tract nucleus, and the ambiguus nucleus (Fliers et al., 1985a). However, Elabd et al. (2014) reported that decreased peripheral OT contributed to reduced muscle regeneration after injury in aged mice.

Human studies investigating age-related changes in OT neurons have been limited to the PVN and SON and do not suggest age differences. For example, there was no age-related change in mean profile area or cell nuclei size in immunocytochemically identified PVN and SON OT neurons in post-mortem brains of 32 individuals ranging from 10 to 93 years (Fliers et al., 1985a, b). Additionally, there was no age-related decline, or sex differences, in the number of PVN OT neurons in post-mortem brains from 20 individuals ranging between 15 and 90 years (Wierda et al., 1991). A more recent study, in 51 younger and 54 older healthy adults, suggested differences in circulatory OT levels, with women in general having higher blood plasma OT levels than men (Plasencia et al., 2019). Although the study did not find a significant age effect for OT plasma levels, numerically younger women had the highest circulatory OT levels and older men the lowest.

Taken together, the endogenous OT system in the PVN and SON appears to remain largely unchanged with age. However, one issue this field of research currently faces is that the brain regions investigated are very specific, rendering the findings limited in scope and generalizability. That is, it is currently not known whether there are age differences in the OT system of the CNS in regions outside of these previously investigated in humans. Knowledge of aging effects in the CNS has become particularly relevant in light of recent evidence of circulatory OT’s role in muscle regeneration in an animal model (Elabd et al., 2014) and possible age-by-sex related differences in circulatory OT in humans (Plasencia et al., 2019).

#### Intranasal Oxytocin Administration in Older Adults

Recent studies have supported age- or age-by-sex differential effects of intranasal OT administration on brain function and social cognition but with some mixed results (Campbell et al., 2014; Ebner et al., 2015, 2016; Grainger et al., 2018a, b; Horta et al., 2018). For example, 24 IU acute intranasal OT, compared to placebo, administration increased functional connectivity strength between amygdala and medial prefrontal cortex (Ebner et al., 2016) and meta mood (Ebner et al., 2015) in older men and younger women, but not older women or younger men. Acute administration of 20 IU intranasal OT, in a study of 68 younger and 68 older adults, improved emotion-recognition abilities in older men compared to placebo and younger adults or older women (Campbell et al., 2014). Grainger et al. (2018a, b), on the other hand, did not see an age-specific effect on theory of mind task improvement after 24 IU intranasal OT administration, but did see sex-specific effects in eye-gaze on a minimal context theory of mind task.

A neuroimaging placebo-controlled study of 46 younger and 48 older adults with 24 IU single-dose intranasal OT administration found age-by-emotion differential effects on functional connectivity during a dynamic facial emotion task but did not find any behavioral effects (Horta et al., 2018). No sex-specific differences were reported for this study. Furthermore, a 10-day 40 IU once daily intranasal OT trial, with 39 older adults, improved emotional well-being, decreased fatigue, and prevented decline in physical functioning, as compared to placebo, on self-report measures (Barraza et al., 2013). However, no other health improvements were observed or sex differences reported in this study. In sum, this growing evidence of variations by age and sex of intranasal OT effects supports the importance of studying the OT system in aging in relation to crucial domains of functioning, including pain.

## Toward a Model of Oxytocin’s Analgesic and Brain Structural Effects in Aging

This focused narrative review has synthesized previously parallel lines of literature toward a novel research proposal. In particular, we document a negative impact of chronic pain on the aging brain and summarize emerging evidence of age-differential effects of intranasal OT, including in the brain, as basis for our proposal that intranasal OT administration may be a viable treatment for pain management in older adults (see Figure 1 *for our Model of Oxytocin’s Analgesic and Brain Structural Effects in Aging*). Age-related changes in the pain experience may be related to altered pain processing in aging and subsequent changes in the brain. These age-related brain changes may play a role in chronic pain susceptibility in older adults, which may be targeted by OT.

As reported above, pain-associated structural alterations of specific brain regions appear to be, at least partially, reversible with pain alleviation (Rodriguez-Raecke et al., 2009). Combining evidence that both endogenous and exogenous OT moderate the pain experience, with recent evidence of neuroplastic effects of repeated OT administration in an animal model (Sánchez-Vidaña et al., 2016), it is plausible that the administration of exogenous OT to the CNS via nasal spray would induce changes in brain structure in the aging brain. These brain changes may include regions involved in pain processing, given the significant overlap between brain structures demonstrating morphological associations with pain and structures that are targets for OT (Figure 2). Based on these considerations we propose that intranasal OT administration constitutes a promising candidate as a pain therapeutic, including in aging. This proposal has critical relevance for future research directions and clinical applications, as discussed in the remainder of the paper (see Box 1).

**Box 1. Selection of promising future research directions and clinical applications in aging.**

□ Effects of endogenous OT system variations (e.g., *OXTR* genotype, *OXTR* gene expression, and blood/saliva/CSF OT levels) on susceptibility for pain chronification.
□ Effects of interindividual variability in neurobiological factors (e.g., *OXTR* genotype and gene expression, brain structure and function, and gonadal hormone levels) on pain alleviation via intranasal OT administration.
□ Systematic investigation of dosage, frequency, and duration of intranasal OT administration for effective management of chronic pain.
□ Safety of intranasal OT administration, also under consideration of drug interactions (opiates and NSAIDs).
□ Interactions of the opioid and the OT systems on pain management and abuse potential.
□ Determination of intranasal OT administration for acute pain alleviation in emergency settings.
□ Age-by-sex differences in intranasal OT treatment response and impact on pain management.

## Considerations for Future Research Directions and Clinical Applications

### Interindividual Variation

Susceptibility to chronic pain development and associated brain structure alterations vary by individual, suggesting that underlying mechanisms beyond injury alone are at play. It is plausible that variance in the endogenous OT system and other pre-existing brain structural variations contribute to individual chronic pain susceptibility profiles. Additionally, variation in sensitivity to pharmacological agents and other treatments may be associated with variance in brain structure (Korgaonkar et al., 2015; Whitfield-Gabrieli et al., 2016), and this structural variability increases with age (Raz et al., 2005; Lindenberger et al., 2013; Papenberg et al., 2015). Future chronic OT administration analgesic trials should include investigations into structural variation of brain regions that constitute strong targets for OT as well as consideration for pain subtype and other population characteristics (e.g., psycho-biological phenotypes, women, socioeconomic status and ethnic/racial groups). Future research in this direction will provide insight into the potential for OT to induce changes in the brain (including morphology) with effects on long-term chronic pain management and will shed light on the mechanisms by which OT exerts its effect and in what contexts.

### Multimodal Neuroimaging

Multimodal neuroimaging will be crucial in detailing some of the relationships proposed in our *Model of Oxytocin’s Analgesic and Brain Structural Effects in Aging*. To this end, future studies could simultaneously employ a variety of neuroimaging techniques, including structural and functional magnetic resonance imaging, diffusion tensor imaging, electroencephalography, and magnetic resonance spectroscopy. Each method contributes to a different, though interconnected, piece of the puzzle furthering the understanding of neurobiological factors involved in the role of OT on pain processing and modulation.

### Dosage and Safety

Short-term controlled intranasal OT administration produced no reliable side effects in doses ranging from 18 to 40 IU (MacDonald et al., 2011). However, systematic dosage studies, including those on intranasal OT safety for long-term chronic pain management in the elderly, are lacking (*but* see Finger et al., 2015; Mameli et al., 2014 *for safety in specific older adult populations*). There is evidence from animal studies that OT may act in a dose-dependent manner on pain (Juif and Poisbeau, 2013). To this end, trials investigating the dosage and safety of intranasal OT for long-term use specifically in older populations, particularly with regards to the potential for medication interactions, are needed. This is especially important as older adults are more vulnerable to medication interactions, also given the increased medical comorbidities associated with advanced age (Wallace and Paauw, 2015). Additionally, standards for appropriate time length for chronic administration of intranasal OT have not yet been established, as the safety and long-term effects of repeated intranasal OT use for pain in humans is yet unknown.

### Routes of Administration

There are various routes of exogenous OT administration in humans, including intranasal, intrathecal, and intravenous (Boll et al., 2017). As OT does not readily pass the blood-brain barrier, intranasal administration is currently the preferred method in clinical trials targeting the CNS (Born et al., 2002; Guastella et al., 2013; Quintana et al., 2018). Intravenous and intramuscular OT targets the periphery and is used to increase uterine contractions during, and control bleeding after, birth. As discussed above, there are differences in OT effects which dependent on the method of delivery (peripheral vs. central) in humans. While these administration routes can result in high blood pressure, heart rate changes, and other side effects (United States Library of Medicine, 2019), intranasal OT administration appears to have a low side-effect profile (MacDonald et al., 2011). However, intranasal administration also increases peripheral OT levels to supraphysiological levels (Leng and Ludwig, 2016); thus, caution must be taken in certain populations (e.g., individuals who are or may become pregnant). Additionally, while animal studies have shown variation in OT response and efficacy between routes of administration, very little is known regarding the differences on pain attenuation between administration routes in humans, thus making this a potential direction for research in chronic pain. *For in-depth reviews on intranasal OT administration* see Leng and Ludwig (2016) and Quintana et al. (2018).

### Concurrent and Adjunct Treatment

Additional considerations when studying OT as an analgesic relate to its potential interactions with concurrent and on-going pain treatments. For example, concurrent NSAID use may interfere with OT’s analgesic effects (Kwong and Chan, 2015), as *OXTR* expression may be, at least partially, driven by inflammatory cytokine presence (Terzidou et al., 2011). Further, OT potentially interacts with the opioid system during pain alleviation (Yang, 1994; Zubrzycka et al., 2005; Taati and Tamaddonfard, 2017) and decreased opioid tolerance in animal models (Kovács et al., 1985; Kriván et al., 1992). In addition, interactions have been found between the OT system with neurotransmitter systems including dopamine (Baskerville and Douglas, 2010) and potentially serotonin (Bakermans-Kranenburg and van IJzendoorn, 2008). This information must be taken into consideration since drugs such as SSRIs or others that act on the serotonergic or dopaminergic systems [serotonin and norepinephrine reuptake inhibitors (SNRIs), etc.] are often used for the treatment of pain or comorbid conditions. Also, other currently unknown drug interactions need to receive particular attention in clinical trials. Given that individuals with chronic pain often use multiple analgesic therapies simultaneously, there are ethical considerations that must be balanced with performing scientifically rigorous research moving forward. This is especially important when there is concurrent use of opioid pain relievers that could be enhanced by OT administration, or other drugs, like NSAIDs, that may render OT ineffective. This emphasizes the need for careful evaluation and research on OT interactions with other commonly used pain treatments.

### Short-Term Acute Pain Alleviation

Given the demonstrated efficacy of acute intranasal OT administration on acute pain sensitivity (Goodin et al., 2014; Rash and Campbell, 2014), clinical trials are needed to determine OT’s potential for short-term or emergency use to reduce pain in instances of acute injury. Along with OT’s acute pain-attenuating effects, intranasal OT has the potential to reduce pain-related anxiety (Singer et al., 2005; Goodin et al., 2014). Future research could include investigations of the potential for intranasal OT to supplement or boost the effects of other pain-relieving drugs in acute clinical settings, while leveraging the potential for anxiety-reducing effects of intranasal OT. However, care must be taken regarding interindividual variation in intranasal OT response and appropriateness of use in certain populations. For example, intranasal OT may have undesirable behavioral effects, such as in-group favoritism and potential bias against out-group members (De Dreu et al., 2011). Older adults may also have compliance issues, such as in instances of dementia.

### Age-by-Sex Differences

Intranasal OT appears to act in a sex-dependent manner, as demonstrated in animal models (Caldwell, 2018) and humans (Lischke et al., 2012; Rilling et al., 2014; Gao et al., 2016). There also is emerging evidence that sex-dependent effects of OT extend to older adults (Campbell et al., 2014; Ebner et al., 2016, 2015; Grainger et al., 2018a). These age-by-sex variations may extend to OT’s efficacy for pain relief. In particular, while a 10-day OT administration in older adults found benefits to self-reported physical functioning (Barraza et al., 2013), a 14-day investigation focused on pain relief for women with fibromyalgia, many of them older, did not find a beneficial effect on chronic pain (Mameli et al., 2014). There are some key differences between these studies (e.g., length of administration and dosage) aside from sex, that could explain the differences in findings. This mixed evidence and current scarcity of previous work highlights the need for additional research to determine under which conditions and for whom intranasal OT may be effective. This combined with evidence that older women are more likely to report experiencing chronic pain (Larsson et al., 2017) calls for future comprehensive investigations of age-by-sex variations in treatment response to intranasal OT administration and its impact on pain management.

## Conclusion

In this review, we integrated parallel lines of research on pain, brain morphology, OT, and aging toward proposition of our *Model of Oxytocin’s Analgesic and Brain Structural Effects in Aging* (Figure 1). Based on evidence of significant overlap between brain regions that have been demonstrated to be involved in pain while, at the same time, constituting key regions of the OT system (Figure 2), our model suggests the use of intranasal OT administration as a viable research direction for chronic pain management, including in older adults. We expect this novel framework to spur future research toward advancement of basic knowledge on the mechanisms underlying OT’s analgesia while informing translational applications to address the monumental impact of chronic pain, particularly in older adults.

## Author Contributions

DL performed the literature search and initial manuscript write-up under close supervision of NE and YC-A. DL, NE, and YC-A worked closely together on the integration and synthesis of the literature, model development, and discussion of future research directions.

## Conflict of Interest

The authors declare that the research was conducted in the absence of any commercial or financial relationships that could be construed as a potential conflict of interest.
